# Good Care during COVID-19: A Narrative Approach to Care Home Staff’s Experiences of the Pandemic

**DOI:** 10.3390/ijerph19042106

**Published:** 2022-02-13

**Authors:** Marleen D. W. Dohmen, Charlotte van den Eijnde, Christina L. E. Thielman, Jolanda Lindenberg, Johanna M. Huijg, Tineke A. Abma

**Affiliations:** 1 Leyden Academy on Vitality and Ageing, 2333 AA Leiden, The Netherlands; eijnde@leydenacademy.nl (C.v.d.E.); thielman@leydenacademy.nl (C.L.E.T.); lindenberg@leydenacademy.nl (J.L.); huijg@leydenacademy.nl (J.M.H.); abma@leydenacademy.nl (T.A.A.); 2 Department of Public Health and Primary Care, Leiden University Medical Center, 2333 ZA Leiden, The Netherlands

**Keywords:** COVID-19, mitigation measures, care homes, wellbeing, care ethics, relational care, narratives

## Abstract

Due to its major impact on Dutch care homes for older people, the COVID-19 pandemic has presented care staff with unprecedented challenges. Studies investigating the experiences of care staff during the COVID-19 pandemic have shown its negative impact on their wellbeing. We aimed to supplement this knowledge by taking a narrative approach. We drew upon 424 personal narratives written by care staff during their work in a Dutch care home during the COVID-19 pandemic. Firstly, our results show that care staff have a relational-moral approach to good care. Residents’ wellbeing is their main focus, which they try to achieve through personal relationships within the triad of care staff–resident–significant others (SOs). Secondly, our results indicate that caregivers experience the COVID-19 mitigation measures as obstructions to relational-moral good care, as they limit residents’ wellbeing, damage the triadic care staff–residents–SOs relationship and leave no room for dialogue about good care. Thirdly, the results show that care staff experiences internal conflict when enforcing the mitigation measures, as the measures contrast with their relational-moral approach to care. We conclude that decisions about mitigation measures should be the result of a dialogic process on multiple levels so that a desired balance between practical good care and relational-moral good care can be determined.

## 1. Introduction

Like many care homes around the world, Dutch care homes for older people have been severely affected by the coronavirus disease 2019 (COVID-19) pandemic. In the Netherlands, within one month after the onset of the pandemic, care home residents represented approximately 10% of all reported COVID-19 cases [1] whilst making up less than 1% of the total Dutch population [2]. After the first wave of the pandemic, from March to June 2020, care home residents were greatly overrepresented in the mortality figures, comprising nearly 46% of all COVID-19-related deaths [3]. During the second wave, from July 2020 to January 2021, this number remained alarmingly high, rising slightly to 50% [4].

The COVID-19 pandemic presented care home staff with unprecedented challenges. The novelty of the disease resulted in great uncertainty about its spread and effects. Since there were shortages of personal protective equipment (PPE), care staff felt pressured to work without protection. This added to the fear of both contracting and spreading the novel coronavirus [5], a fear that became reality, as many residents were infected with and died from COVID-19. It was not only the disease itself, but also the related mitigation measures that were challenging for care staff. For instance, they were required to enforce room isolation for residents suspected of having a COVID-19 infection, as well as a temporary visiting ban, excluding residents’ significant others (SOs) and other informal caregivers from the care home. 

In the past two years, many studies have been published about the experiences of care staff during the COVID-19 pandemic. The results show that doctors and nurses are traumatized from working on the front line [6,7]. They were confronted with dire situations and dilemmas due to the limited capacity of the health system and limited resources [8,9], all the while balancing their duty to care with their own safety and that of their families [6]. In several instances, staff shortages, limited resources and the following of COVID-19 mitigation protocols resulted in negative medical outcomes and sometimes even death for those receiving care [10]. Moreover, many care workers on the front line died as a result of COVID-19 infection [11]. In addition, the COVID-19 pandemic amplified existing disparities in the care sector. Firstly, COVID-19 mitigation measures reinforced an existing valuation of *cure* over *care,* as short-term care settings were given priority over long-term care settings with regard to COVID-19 testing and the distribution of PPE [12,13,14]. Secondly, freelance care workers in care homes were exposed to more dangerous situations than permanent staff, as they had less access to PPE and were more often required to work risky shifts [15]. The challenging circumstances that care staff faced during the COVID-19 pandemic are reflected in the results of a systematic review investigating the impact of COVID-19 on care staff’s mental health. The results included increased depressive symptoms, anxiety, stress and poor sleep quality (for detailed information see [16]). Similar results have been found for Dutch care home staff in particular, with reports of increased work and emotional loads and decreased wellbeing since the onset of the COVID-19 pandemic [5,17].

The studies published about experiences of care staff during COVID-19 thus far have mainly relied on surveys and interviews that use pre-defined topics. We aim to supplement the knowledge gained from these studies by taking a more explorative approach. In January 2020, we started an action research project collecting narratives from care home staff about their daily experiences providing care. Care home staff were free to share narratives about whichever topic in their work was important to them and they continued to share these narratives throughout the COVID-19 pandemic, allowing us to collect a large amount of data foregrounding care home staff’s own perspectives during this time. To our knowledge, there is no prior study that has taken this approach. In addition, the research methods used to investigate the experiences of care staff during the pandemic have thus far been mainly retrospective. Our study aims to capture care staff’s lived experience as closely as possible by asking them to share their daily experiences during their work shift soon after the fact. 

An explorative approach centered on the emic perspectives of care staff could help us gain a deeper understanding of the experiences of care staff during the COVID-19 pandemic. Further knowledge about this is needed to mitigate the negative effects of the pandemic on care staff, especially in the long run. Previous studies have highlighted the risks and prevalence of moral injury and compassion fatigue for care staff due to the dire situations and moral dilemmas they encounter during the COVID-19 pandemic [18,19,20]. Dilemmas in this context do not only revolve around life and death, but also around everyday ethics, as was found in an international study on the ethical challenges faced by care staff during the COVID-19 pandemic [21]. By taking an explorative approach, we may gain better insight into the dilemmas care staff encounter during the COVID-19 pandemic, perhaps especially those that occur in everyday work. These insights can then serve as a knowledge base to counter moral injury and compassion fatigue for care staff. In addition, learning more about the experiences of care staff during the COVID-19 pandemic can help us prepare for any similar scenarios in the future, as the intention of care staff to care in a public health crisis has been linked to their experiences during previous public health crises [22]. 

We aim to contribute to existing knowledge by answering the following research question: Which experiences did Dutch care home staff describe in their narratives during their work shifts in the COVID-19 pandemic? We draw upon original data from 424 brief personal narratives, written by care home staff members during the first and second waves of the COVID-19 pandemic in the Netherlands. Within these narratives, it is shown that care staff have a relational-moral approach to care and that the COVID-19 mitigation measures obstruct their efforts to deliver care in the relational-moral sense.

## 2. Materials and Methods

### 2.1. Ethical Approval

This study was reviewed and declared not subject to the law on research involving human subjects by the Institutional Review Board of the Medical Ethical Committee Leiden-Den Haag-Delft for observational studies and was registered under number N20.095. The protocol was assessed and considered compliant with scientific due diligence. 

### 2.2. Context of the Study

Data collection for this study took place as part of a larger study examining the possibilities of using narratives about personal experiences with care to account for its quality. The study took place from January 2020 to December 2021 on the psychogeriatric ward of two care homes in the Netherlands. During this time, care staff captured many brief personal narratives about whichever topic in their work was important to them using the digital application SenseMaker^®^ [23]. Consequently, we collected many narratives during the COVID-19 pandemic in the Netherlands. These narratives took place in the context of COVID-19, but their content was not restricted to COVID-19.

### 2.3. Design

The larger study had an action research design and was conducted in close collaboration with the stakeholders in practice. The sub-study presented here had a qualitative design, in which we analyzed narrative data through open coding.

### 2.4. Sampling

Sampling was restricted to the two care homes partaking in the larger study. For the current study, only data from one of the two care homes was included. The care staff in this care home continued to share their narratives throughout both the first and the second waves of the COVID-19 pandemic. Care staff in the other care home stopped sharing narratives because of the increased workload due to the COVID-19 pandemic, resulting in not enough data for analysis. 

The psychogeriatric ward included in the study had two units, separated by a hallway. Both units had individual rooms for each of their 13 residents and a main living room. There were two shifts a day and three care workers present on each unit during a shift. These were vocational nurses and certified nursing assistants. During some shifts, there were also an activity worker and a hostess present. The care staff working on the ward were predominantly female. The residents living on the ward all had a diagnosis of dementia and were generally in an advanced state of the disease. They required complex care due to multimorbidity, low mobility and frailty, and their dementia brought about cases of challenging behavior such as shouting, wandering and aggression. During the studied period, there was one reported case of a COVID-19 infection on the ward.

During the study, the entire care team on the ward had access to the digital application SenseMaker^®^. Six of the 25 team members were enthusiastic about using it regularly and partaking in research activities. Among these six active participants were two activity workers and four vocational nurses. These participants were between 35 and 57 years old and were all female. The six individuals were also the ones who received coaching (see Section 2.6). The digital application remained open to other care team members, who used it sporadically. Employees of the care home organization who were not part of the care team of the specific psychogeriatric ward were excluded from participation.

### 2.5. Data Collection and Materials

Data was collected using the SenseMaker^®^ application, a distributed ethnography tool suitable for complex environments [23]. Participants had access to this application via smartphone, computer and tablet. Participants captured their narratives as a written answer to the following open-ended questions: ‘What did you do or experience during your shift that stuck with you? What happened and how did this affect you?’ Participants were free to share narratives about whichever topic or experience was important to them, not restricted to COVID-19. Within the narratives, care staff described a specific experience they had, in about 3–10 sentences. They described what happened and how this affected them and others. They captured their narratives during their work shifts or shortly after. The use of this explorative method contributed to the collection of data that closely resembled participants’ lived experiences. 

In total, 723 narratives were captured by care staff during the course of the project. To answer the research question, only narratives submitted during the first (March through June 2020) and second (July 2020 through January 2021) waves of the COVID-19 pandemic in the Netherlands were included. All narratives shared during this period were included, from the six active participants as well as from the other care team members. This led to the inclusion of 424 narratives. 

### 2.6. Procedure

Over the course of four months, the six active participants received individual coaching by authors MD and CE in becoming aware of their experiences and capturing their narratives of them. The coaching consisted of four exercises carried out by the participants, each followed by a telephone conversation with either MD or CE to discuss the exercise. Participants’ feedback and remaining learning needs served as input for the content of each following coaching round. 

In this coaching process, we followed an action-reflection learning approach [24]. Participants worked together in small groups to develop the skills necessary for this project and to reflect on the situations they encountered in their work. For the first exercise, participants were asked to take a picture of (a moment with) a resident that, to them, said something about that resident specifically. For the second exercise, participants were asked to reflect on a written narrative of a fictive experience and to discuss whether the experience was described in a useful and complete manner. The third exercise encouraged participants to learn from each other’s experiences. Participants reflected on narratives shared by their colleagues and discussed how sharing narratives could serve as a source of inspiration in their work. The fourth exercise focused on becoming more aware of one’s own qualities. Participants discussed which of their qualities they utilize to contribute to positive experiences for a particular resident. After each coaching round, a newsletter with reflections on the exercise and a selection of that month’s captured narratives was shared with the entire care team. 

There were no requirements as to the amount of narratives care staff had to share. We stimulated the capturing of narratives by verbally encouraging the care staff, by being regularly present on the ward (outside the period of the visiting ban), by distributing posters and newsletters around the ward and by celebrating milestones. Participants also engaged in peer-to-peer coaching, encouraging their colleagues to capture narratives. 

### 2.7. Analysis

Given the explorative nature of our study, and our goal to follow the content of the experiences of the participants, the narratives were analyzed using thematic analysis [25] by the authors CE and CT independently. MAXQDA (version 20) qualitative data analysis software was used for analysis. Through coding, the authors CE and CT identified themes and patterns in the data. The coding authors then compared and discussed the codes and after discussion merged them into one code book. When data was coded differently, the codes were discussed until a consensus was reached between the authors CE and CT. In addition, the coding authors indicated in which narratives the participants’ work during COVID-19 was described in general terms and in which narratives they shared the specific impact of COVID-19 on their work. These codes were compared and discussed with MD, resulting in a final set of general narratives and COVID-19 specific narratives. Next, the process of constant comparison was initiated [26], in which authors CE, CT and MD compared codes to discover relations and patterns within the general themes. In this phase, several meetings were held between the first author and the coding authors CE and CT. The first meetings were focused on descriptive findings, whereas the following meetings focused on identifying relations and patterns within the general themes. 

The narratives presented in Section 3 have been translated from Dutch to English by the authors, anonymized and edited for clarity.

## 3. Results

From the 424 narratives care staff shared during their work shifts in the COVID-19 pandemic, the vast majority (362) are about experiences that took place during the pandemic but were not explicitly related to COVID-19. From these narratives, we gain insight into care staff’s general approach to care in times of COVID-19. Below, we first describe care staff’s approach to care, based on these general narratives. Thereafter, we describe the specific impact of COVID-19 on care staff’s approach to care, the wellbeing of residents and their own wellbeing, based on 62 narratives specifically about COVID-19 and related measures. An overview of results can be found in Table 1.

### 3.1. Care Staff’s Approach to Care during COVID-19

The wellbeing of residents plays a central role in the narratives that care staff shared about their work during the COVID-19 pandemic. Specifically, the narratives show a strong focus on residents’ wishes and desires, rather than their needs and limitations. Care staff described how they offered small daily activities to residents and how this lightened up their mood. Care staff also described how they used knowledge of the resident’s identity to tailor these activities to personal wishes and desires.

*“A resident’s happy face during the daily care. I know that this resident used to teach English and loves music very much. During the daily care, I started talking English to her and my colleague joined in. We spontaneously started singing a song in English and the resident sang along at the top of her lungs. After the song, she said how much she liked it. A wonderful and beautiful experience, so much happiness.”*

A relational approach to care is important for getting to know the resident’s identity. In addition, the narratives show that care staff see their relationship with residents as inherently valuable as well. They wrote about one-on-one moments with residents and described these to have had a direct positive effect on the wellbeing of both residents and care staff.

*“Smoking a cigarette together with a resident of whom I know enjoys this enormously. A moment with just the two of us, a quiet chat. Enjoying being outside for a moment, away from the rest of the ward. A moment of one-on-one attention during the day, moments to cherish. I enjoy seeing him enjoying himself in these moments. He also says this himself, very beautiful and inspiring.”*

Furthermore, care staff described feelings of frustration and powerlessness when they encountered difficulties in easing a resident’s agitation, sadness or aggression. These types of narratives emphasize the complexity of caring for and contributing to the wellbeing of people with dementia.

*“It’s hard when you don’t understand someone’s behavior and have to deal with their frustrations every day. You want everyone to enjoy themselves and be well, but there are some aspects over which you have no control and I find that difficult at times.”*

Within the narratives, care staff described the value of the relationship between residents and their SOs for the wellbeing of residents. To enhance residents’ wellbeing, care staff try to facilitate this relationship, both practically, e.g., ensuring that the resident is out of bed and dressed when SOs arrive to visit, and emotionally, e.g., supporting SOs in making contact with their loved one who has dementia.

*“A resident’s wife asked me for additional guidance during her visit. I explained to her that her husband just likes to sit and listen to his wife talking. Indeed, the resident sat and listened with a smile. His wife told me she felt so much more at ease now that she had seen her husband smile during the visit.”*

The narratives show that care staff desired appreciation from SOs for the work they do. On the one hand, care staff shared narratives about how happy they were when SOs expressed their gratitude. On the other hand, they also shared narratives about how it affected them when SOs were dissatisfied with the care provided. Furthermore, care staff described how they handled dissatisfaction from SOs. They tried to resolve it by engaging in conversation and explaining their way of working. Generally, these conversations ended with an agreement to approach things differently next time, whether it be by the care staff or by the SO.

*“A resident’s wife came to me during the physical exercise hour. She was upset that she was not informed about this activity and she would like her husband to participate. I told her that her husband always participates when there are no visitors. I had also told her twice before that there was an exercise hour every Thursday afternoon. I let her talk and cry. After she had calmed down a bit I told her that I would inform her daughter when the exercise hour would take place and I would put it in the resident’s agenda. She was happy about this and said that she knows I am doing my best, but that she hates it when her husband is not involved and just sits there by himself. I can understand that. I put the appointment in the agenda for next week and informed her daughter by email. These kinds of conversations are quite difficult but also quite exhausting because it never seems to be good enough for this family.”*

### 3.2. The Impact of COVID-19 

From the 62 narratives about COVID-19 specifically, the vast majority (59) were about the impact of the mitigation measures. The other three were about the impact of the disease itself. Temporally, the 62 narratives specifically about COVID-19 were concentrated on changes in the mitigation measures. The mitigation measures care staff described most were: (i) ward isolation for all residents; (ii) room isolation for residents suspected of having a COVID-19 infection; (iii) wearing PPE; (iv) the visiting ban and (v) required supervision during visits after the ban was lifted. Enforcing these mitigation measures was part of care staff’s duties during the COVID-19 pandemic. Care staff described the impact of the mitigation measures on their approach to care, the wellbeing of residents and their own wellbeing. These findings are presented below.

#### 3.2.1. COVID-19 Mitigation Measures Alter Care Staff’s Approach to Care

Care staff’s approach to care was impacted by COVID-19 mitigation measures in both the first and second waves of the COVID-19 pandemic in the Netherlands. 

Within the narratives, care staff wrote about having to turn down appeals to care and support from dependent residents, hindering them from meeting residents’ wishes and desires. In addition, there were several cases in which the mitigation measures required care staff to actively contribute to negative experiences for residents, for instance when they had to physically guard residents from leaving their room when in isolation.

*“A resident grabbed my hand and asked me not to leave her. She wanted to come with me to the other ward. Unfortunately that is not possible during corona. Such a difficult moment. I truly had a hard time having to leave her behind!”*

With everyone isolated on the ward, care staff described high tensions between residents and how they had to serve as mediators. They shared how residents were quickly annoyed with each other and how small incidents led to verbal aggression, threats and physical altercations. Due to the residents’ dementia, keeping the peace by explaining the situation to residents did not always work.

*“Mrs. Schwarz is not in a good mood. She is short-tempered. One of the residents is yelling ‘help me, help me’ and this has been going on for a long time. Mrs. Schwarz rolls towards her. ‘Shut up, stupid woman! If you don’t, I’ll slap you or throw you out!’ She has a threatening attitude. I addressed her on her behavior, but 10 min later the above-mentioned repeated itself.”*

Furthermore, the narratives indicate that the mitigation measures negatively impacted on the relationships between care staff and residents. Firstly, care staff described how wearing a face mask formed a physical barrier to making contact with residents. Moreover, they wrote that residents were confused as to why care staff were wearing masks and why they were refusing to take them off.

*“Several times a day a resident asks me: ‘take off that mask, don’t be silly. I think it’s nonsense, I can’t see you now.”*

Secondly, care staff wrote that residents were frustrated by the limited contact they could have with their SO’s because of the visiting ban. Since care staff members were the ones required to enforce the visiting ban, residents had difficulties understanding why this measure was taken and were prone to blame the care staff personally.

*“A resident is standing at the window. Her husband has come to wave to her from outside. As a result of the corona restrictions, no visitors are allowed. The resident gestures to her husband: ‘come upstairs.’ I explain to her that that’s not possible. ‘Then I’ll go to him.’ I tell her that’s not possible either. ‘You are so mean’, she tells me.”*

Regarding the contact between residents and SOs, the narratives show that care staff’s facilitation changed from supportive to crucial during the visiting ban. Without the help of care staff, no contact would have been possible at all between those inside and those outside the care home. Care staff wrote about enabling remote contact between residents and SOs by helping residents to video call with their SOs and by guiding residents to the window when their SOs were standing on the terrain of the care home, wanting to wave to their loved one inside. In addition, there were instances where care staff took on part of the role that the SO usually had.

*“A resident asked her daughter: can you bring me some eel? Daughter is not allowed to do this because of COVID. Therefore, I took it upon myself to bring some eel for her. The resident was totally surprised and became emotional. She kissed and hugged me. We sent a picture to her daughter.”*

Meanwhile, care staff’s own contact with residents’ SOs took on a predominantly negative tone. Due to COVID-19, care staff often had to be the bearer of bad news towards SOs, whether it was by communicating new restrictions or by enforcing existing ones. Care staff wrote how they were apprehensive to convey these messages, as they did not want to hurt SOs and were anxious about their reactions.

*“There are visitors in the living room, even though that is not allowed during this corona time. A colleague is afraid to point out the rules to them and asks if I will do it. With jitters in my stomach I approach the family. I ask them to leave the living room. They react somewhat resentfully and choose to break off the visit. The residents at the tables find it ridiculous and are angry. The cheerful mood has clearly turned. The resident for whom the visitors stopped by is shouting that this is no way to treat people.”*

As the first point of contact, care staff were frequently confronted with SOs’ frustration over the mitigation measures. They wrote about SOs expressing their grief over the measures, urging them to relieve the measures, blaming them for enforcing the measures and reacting angrily when they held them to the measures.

*“I received a call from the family of a resident. I’m getting a lot of blame. They don’t agree with the visiting rules. They think it’s ridiculous. ‘Grandma doesn’t need to be accompanied, can’t she just visit him alone?’ It’s not allowed, I tell them. Besides, there is a supervisor in the visiting area, supervising everyone. We get accused of giving grandma a second heart attack. It feels like they’re blackmailing us.”*

Furthermore, the narratives show that care staff strictly adhered to the mitigation measures, despite the challenges this posed (e.g., dissatisfaction from SOs). Care staff’s adherence to the rules shows that they saw no room for conversation with residents and SOs over the measures. This differed from their usual approach to care, in which they engaged in conversation to dissolve dissatisfaction, as shown under Section 3.1. 

#### 3.2.2. Residents’ Wellbeing: Restricted Freedom and Limited Contact with SOs 

Care staff’s narratives about the impact of the mitigation measures on residents’ wellbeing focused strongly on two mitigation measures from the first wave of the COVID-19 pandemic in the Netherlands: the visiting ban and the isolation of residents on the ward. Another mitigation measure that was often described in this context was room isolation for residents suspected to have a COVID-19 infection, which was a measure in place in both the first and second waves. 

The narratives show that residents’ restricted freedom due to ward and room isolation had a negative impact on their wellbeing. Care staff described how the isolation caused confusion, agitation, sadness, anger and despair.

*“Mr. Silva is isolated in his room due to suspected COVID. I am accompanying him in his room this morning. I am in protective gear, safety goggles, face mask. Mr. Silva is very emotional. He has lost all hope. He misses his wife and he is in pain. He rolls to the window and wants to open it. He tries to stand up and says: I’d rather end things. This gets to me. Luckily, his test result is negative and the room isolation is lifted.”*

Due to the visiting ban, there was only limited contact between residents and their SOs, which care staff described as having a negative effect on residents’ wellbeing. Residents missed their SOs and did not understand why they did not visit anymore. Care staff indicated the restrained contact with SOs led to increased apathy, agitation and challenging behavior in residents.

*“Mr. Spears tells me he is in a bad place. He is not seeing his loved ones. Not his wife, not his children. I explain to him that visitors are not allowed right now. He says that it’s ridiculous.”*

To remain in touch, SOs sought other ways of making contact during the visiting ban. The most common of these were conducting video calls and standing on the terrain of the care home, waving to their loved ones inside. Care staff described the positive effect of remote contact on residents’ wellbeing.

*“During the outdoor performance Mr. Simon was partying hard. After two gloomy days, it was beautiful to see how he was uplifted by the music and his wife and son who were downstairs dancing along! Really wonderful to see!”*

However, the narratives also show that remote contact was no replacement for face-to-face contact. Remote contact was one-dimensional, did not allow for intimacy and was sometimes mentioned to be confusing for residents. 

#### 3.2.3. Care Staff Wellbeing: Experiencing Dire Situations and Internal Conflict

Care staff were confronted with dire situations on the ward in both the first and second waves of the COVID-19 pandemic. Their own wellbeing appeared to be linked to residents’ wellbeing, as they described how the negative effects of mitigation measures on residents in turn affected them.

*“I was emotionally very affected by the despair of a resident who had to be isolated in his room. I found it difficult to be confronted with such visible suffering. Especially since there wasn’t much I could do besides lend an ear and be physically present. I sat in the office and cried for a while afterwards.”*

Furthermore, care staff described how having to enforce mitigation measures led to internal conflict. The narratives show that care staff experienced large contrasts between what they were required to do (enforcing and adhering to mitigation measures) and what was in their hearts (contributing to positive experiences for residents).

*“I accompanied a resident to the first visit from his wife [since the visiting ban was lifted]. They have to stay 1.5 m apart. They can’t touch each other. No hug, no kiss. Not even when saying goodbye. Both the resident and his wife are having a hard time with this. His wife calls it degrading. I find it so sad it has to go like this. I would really love to just allow it.”*

## 4. Discussion

By taking an explorative approach in which care staff were free to share narratives about whichever topics in their work were important to them, we have gained an insight into their approach to care during the COVID-19 pandemic in general terms, as well as into the specific impact of COVID-19 on their approach to care, the wellbeing of residents and their own wellbeing. 

The results show that within care staff’s narratives, residents’ wellbeing was the main focus during the COVID-19 pandemic. Furthermore, care staff see their relationships with residents as important way in the context of contributing to their wellbeing. As a consequence, they invest in getting to know the residents to attune care to their personal wishes and desires, although this is at times challenging due to the complexity of caring for people with dementia. Moreover, care staff aim to facilitate the relationships between residents and their SOs, as they see this as an important influence on residents’ wellbeing. Within their own relationship with SOs, care staff engage in dialogue to come to a mutual understanding of good care, whilst desiring appreciation from SOs for the work they do.

The way of working that is foregrounded in care staff’s narratives is in line with the relational-moral approach to good care in care ethics, which states that the definition of good care is shaped in and by the relationship between the caregiver and care receiver. In good care, it is not the rules and procedures that are most prominent, but the nature of the caregiver –care receiver relationship. Accordingly, Tronto [27] states that good care is a two-way affair: it is the outcome of a dialogic process between caregiver and care receiver and adjusted to personal needs, wishes and desires [28]. Our data clearly shows that the relational approach does not only concern the resident–care staff relationship, but extends to SOs in a reciprocal interaction of appreciation, value and meaningful engagement.

In 62 narratives, care staff explicated the impact of COVID-19 on their experiences, predominantly in terms of the impact of the COVID-19 mitigation measures. Due to the effects of these measures on care staff’s approach to care and residents’ wellbeing, the mitigation measures can be seen as obstructions to good care in the relational-moral sense in several ways. 

Firstly, the mitigation measures limited care staff’s ability to attune care to residents’ personal wishes and desires, and sometimes even required care staff to contribute to negative experiences for residents. This was in stark contrast to their preferred approach to care. Moreover, the mitigation measures formed a serious threat to residents’ autonomy by isolating them from the outside world [29,30]. The fact that the mitigation measures focused on adding days to residents’ lives rather than adding life to residents’ days, shows the dominancy of thinking about good care in a practical sense rather than in a relational-moral sense. Indeed, research shows that in situations of environmental pressure, practical care often prevails over relational-moral care [31].

Secondly, the mitigation measures put pressure on the relationship between care staff and residents. Care staff had to enforce the mitigation measures, which frustrated residents. Moreover, care staff frequently had to turn down appeals for care and support from residents. Importantly, a relationship of trust is a requirement for relational-morally good care [32]. Tronto [27] describes how trust between caregiver and care receiver is developed in a cyclical process of four phases. In the first phase, the caregiver is attentive and recognizes the need for care in the care receiver. In the second phase, the caregiver accepts the responsibility to meet the identified care needs and determines how to do this. The third phase consists of the caregiver using her expertise to directly meet the care needs. In the fourth and last phase, the care receiver responds to the received care, to which the caregiver is once again attentive, constituting a cycle. It is plausible that the imposing of mitigation measures diminished the trust residents had in their caregivers, especially since it was often unclear to residents why care staff were acting in this way. The narratives show that the mitigation measures mainly intervened in the second and third phase of Tronto’s cyclical care process. Caregivers were still attentive to residents’ needs but were no longer able to take responsibility and actions to meet these needs, causing moral distress [18,19,21]. Instead, they had to take responsibility for a different aspect of care (i.e., physical health and safety), which did not directly match residents’ explicit wishes and desires. The residents’ responses to the safety measures (i.e., Tronto’s fourth phase) confirms this mismatch, as their sadness, frustration, desperation and challenging behavior increased. 

Lastly, the safety measures put a strain on the contact between care staff and SOs, as communication over the mitigation measures resulted in their interactions becoming primarily negative. In addition, the foregrounding of life itself over living, and with that practical care, left no room for dialogue. This differed greatly from care staff’s usual way of engaging with SOs, which involves conversation and compromise, as they describe in their narratives. As the care staff put forward, they have a relational-moral approach to care, meaning they view good care as an intersubjective understanding reached through a dialogic process. Therefore, not being able to align value perspectives about the mitigation measures forms an obstruction to delivering relational-morally good care. The strained relationships between care staff with SOs during the COVID-19 pandemic is incongruent with previous findings showing that people cooperate rather than compete in times of crisis, but may be explained through an important determinant of this cooperation: an emerging sense of identity [33]. The mitigation measures emphasized the boundaries between those inside and those outside the care home, creating an ingroup and an outgroup. As such, the shared identity that emerged within the care home may have differed notably from that outside the care home, hampering cooperation.

The finding that mitigation measures posed obstructions in terms of a relational-moral approach to care is in line with findings from an international study into the experiences of social workers during the COVID-19 pandemic [21]. The study found that social workers had to work harder to establish and maintain relationships of trust with those receiving care, whilst only being partially able to meet care needs.

Importantly, the narratives also show that being confronted with dire situations due to the COVID-19 mitigation measures had a negative impact on the wellbeing of care staff themselves. This is in line with several studies showing an increase in emotional load, depression, anxiety and stress for care staff due to COVID-19 [5,17]. Furthermore, we found that having to enforce mitigation measures led care staff to experience internal conflict. The internal conflict described by care staff is in line with a recent finding showing that care staff experiences feelings of guilt when enforcing the mitigation measures [34]. The internal conflict experienced by care staff may be viewed in terms of moral distress. As practical care was foregrounded over relational-moral care, care staff had to repeatedly take actions that violated their moral code. Ultimately, this could result in moral injury, a risk that several studies have highlighted [18,19,20].

The COVID-19 pandemic is and has been an evolving situation. Given its temporal dynamics, we compared narratives from the first and second waves of the COVID-19 pandemic in the Netherlands. There were no specific temporal changes in the impact of COVID-19 on care staff’s approach to care other than changes in mitigation measures. Although different measures presented different challenges, they each presented challenges for the triadic relationship between care staff, residents and SOs. Regarding the impact of COVID-19 on the wellbeing of residents, the narratives centered around mitigation measures promoting isolation, which were stricter in the first wave of the COVID-19 pandemic in the Netherlands than in the second wave. Narratives about the impact of COVID-19 on the wellbeing of care staff did not present specific temporal changes. Care staff wellbeing was affected by the negative effect of mitigation measures on residents’ wellbeing and by the internal conflict they experienced when enforcing mitigation measures; both factors were present throughout the first and second waves of the COVID-19 pandemic in the Netherlands.

### 4.1. Strengths and Limitations

The two strengths of our study are the explorative approach, in which care staff were able to share narratives about whichever topic was important to them, and the capturing of data shortly after the experience took place. By taking such an approach, we were able to capture care staff’s lived experiences during the COVID-19 pandemic as closely as possible. In addition, the narrative approach highlighted the impact of COVID-19 in terms which cannot be expressed by numbers. Future studies may include the perspectives of care staff to a further degree by involving them in the process of data interpretation as well. 

One possible limitation of our study is that we have no way of comparing the narratives shared during the COVID-19 pandemic with narratives outside of the context of COVID-19, and so we cannot be sure whether or not COVID-19 affected care staff’s approach to care in more ways than they explicitly stated by themselves. The strong focus on residents’ wellbeing may have been a way to elicit positive emotions, which is an effective coping strategy in times of stress [35]. The desire for appreciation from residents’ SOs may have also been a result of the extra pressure COVID-19 put on care staff as intermediaries and bridges to the outside world. 

Another limitation is that our study had a small sample size. Narratives from care staff stemmed from one psychogeriatric ward only. When it comes to the generalizability of our findings, there are several factors to consider. Firstly, care staff on the ward included in the study worked according to the Enjoying Life approach, a narrative approach focused on residents’ personal wellbeing and desires rather than their needs and limitations [36]. The impact of the COVID-19 pandemic on care staff’s approach to care may be different for care staff working on wards where the approach to care differs to begin with. Secondly, there was one case of COVID-19 on the ward included in the study during the studied period, reported on the last day of the second wave (31 January 2021). A different image of the impact of COVID-19 may have emerged on wards where care staff experienced more COVID-19 infections and COVID-19-related deaths. Thirdly, individual differences are likely to play a role in one’s wellbeing and approach to care during the COVID-19 pandemic. These may include socioeconomic status [37], job type (e.g., a nurse whose daily tasks focus on medical procedures or an activity therapists whose work is focused on residents’ mental wellbeing) [38], fear of COVID-19 [39,40] and level of resilience [41]. Comparing narratives between the two care homes included in the larger study may have provided us with a more diverse understanding of the experience of care home staff during the COVID-19 pandemic. However, data from the second care home was too limited for analysis. 

Lastly, we used the SenseMaker^®^ application in a longitudinal fashion, while it was developed to be used in a cross-sectional one. SenseMaker is suitable for gaining an understanding of complex systems, which suits the context of the care home better than research methods that assume linearity. Testing the applicability of SenseMaker^®^ to collect and use narratives about personal experiences with care to account for its quality was part of the goals of our larger study. Whilst we worked together with the developers of SenseMaker^®^ to adjust the application to this goal and context, the method has not yet been validated for longitudinal use. For more information on SenseMaker^®^ as a research tool see [23]. 

### 4.2. Implications

Dichter and colleagues [29] described the importance of balancing infection prevention and person-centered care during the COVID-19 pandemic to maintain the wellbeing of older people living in German care homes, as did Verbeek and colleagues [42] in the Dutch context. By stemming from the direct experiences of care staff, our findings underline and stress the importance of finding this balance. 

Furthermore, our findings underline the importance of a relational-moral approach for the wellbeing of all involved in the triad of care staff–residents–SOs. This finding is in line with developments in the Dutch long-term care sector, as the focus has shifted over the past few years from physical health, protocols and evidence-based practice to relationships, wellbeing and attuning care to personal wishes and desires [43]. Our findings also show that care staff value this relational-moral approach to care, and yet they had to adhere to COVID-19 mitigation measures that foregrounded practical care. Finding the right balance in such a situation is extremely difficult. The notion of democratic care may offer guidance in this context [28,44]. Democratic care foregrounds an ongoing dialogic process within the triad of care staff–residents–SOs. Through this dialogic process, all parties collaboratively determine what good care is. In a previous study, we extended the notion of democratic care to include dialogue about good care on multiple levels: on a micro-level (between care staff, residents and SOs), on a meso-level (between care staff and managers) and on a macro-level (for internal policy and the reporting of quality of care to supervisory bodies) [44]. Mitigation measures and their implications for good care may be discussed and decided upon through an ongoing dialogic process between those involved on multiple levels. In this process, those in power should actively create space for the voices of people in vulnerable situations, as this constitutes the democratic potential of a care home [44].

However, creating room for such a dialogue in a crisis situation has been proven difficult. In the Netherlands, many decisions about handling the COVID-19 pandemic were taken in a top-down manner. Consequently, our study shows that care staff encountered situations of moral distress, where the actions they had to take (i.e., mitigation measures) violated their moral codes. In turn, care staff felt that they had no room to engage in a conversation about good care with residents and SOs. In a previous publication, we highlighted the importance of a structure and a culture for engaging in dialogue to enhance the democratic potential of care homes. Ensuring a structure and a culture of listening to a variety and voices and discussing differences under normal circumstances may help create more space for dialogue on multiple levels in a crisis situation as well. 

Furthermore, our findings underline the importance of ongoing mental support for care staff during the pandemic. This is not only needed to protect their wellbeing, but also to retain care workers in the care sector. The Dutch care sector has been experiencing a large outflow of workers regardless of COVID-19, mainly due to lack of opportunities for personal development. The resulting personnel shortages lead to high levels of time pressure and workload, leading others to leave the sector as well [45]. The extra pressure COVID-19 puts on care staff has increased the already problematic outflow of care workers from the sector [45]. Our findings show that measures to retain and attract care staff may focus on creating more opportunities to deliver care in a relational-moral way, as care staff views this as fundamental to good care. The notion of democratic care as described above, in which dialogue between those involved in the care process plays an important role, may offer further guidance in this context.

## 5. Conclusions

In the study presented here, we have shown that residents’ wellbeing has been Dutch care home staff’s central focus during the COVID-19 pandemic. They aimed to achieve this by forming and maintaining relationships within the triad of care staff–residents–SOs. Their way of working is in line with relational-moral approach to good care in care ethics, where good care is a two-way affair: its definition is the outcome of a dialogic process, shaped in and by the caregiver–care receiver relationship [27,28]. Importantly, we find reciprocal appreciation is essential in the triadic relationship between care staff, residents and SOs and therewith extend Tronto’s caregiver–care receiver relationship to include SOs. 

When it comes to the impact of COVID-19, care staff are primarily concerned with the mitigation measures and moral distress related to these measures, rather than the disease itself. We have found that care staff see the mitigation measures as obstructions to good care, as they harm residents’ wellbeing, limit the ability of care staff to contribute to residents’ wishes and desires, damage the care staff–resident relationship in terms of trust, sour the contact between care staff and SOs and leave no room for dialogue about the definition of good care in different situations. 

In addition, we have shown that COVID-19 mitigation measures have a negative impact on care staff’s own wellbeing. Enforcing mitigation measures leads care staff to be confronted with dire situations and emotionally charged responses from both residents and SOs. In enforcing the mitigation measures, care staff experience internal conflict, as what they are required to do differs from what they see as good care. 

Our findings underline the importance of finding a balance between good care in a practical sense and a relational-moral sense. Prioritizing life over living itself, as happened during the COVID-19 pandemic, is often not in line with care staff’s, residents’ and SOs’ views on good care. Our way of handling COVID-19 in care homes should therefore be a process of ongoing dialogue on multiple levels about the type of good care those involved value most. Care home organizations should acknowledge a variety of voices and ensure that there is a structure and culture in place which can accommodate the discussion of differences. Those in power should actively make space for the voice of people in vulnerable situations, on multiple levels.

## Figures and Tables

**Table 1:** Overview of themes emerging from care staff’s narratives.

Emerging Themes	Emerging Sub-Themes	Relationships Involved
Care staff’s approach to care during COVID-19	Focus on residents’ wishes and desires	care staff–resident
	Knowledge of the resident’s identity to tailor care	care staff–resident
	Care staff–resident relationship as inherently valuable	care staff–resident
	Complexity of caring for people with dementia	care staff–resident
	Valuing the resident–SO relationship	Resident–SO
	Facilitating the resident–SO relationship	care staff–resident–SO
	Need for appreciation from residents’ SOs	care staff–SO
	Conversation about dissatisfaction from residents’ SOs	care staff–SO
	Focus on residents’ wishes and desires	care staff–resident
	Knowledge of the resident’s identity to tailor care	care staff–resident
	Care staff–resident relationship as inherently valuable	care staff–resident
	Complexity of caring for people with dementia	care staff–resident
Mitigation measures alter care staff’s approach to care	Turning down appeals to care and support	Care staff–resident
	Actively contributing to negative experiences	Care staff–resident
	Tensions between residents on the ward	Resident–resident
	Mediating conflicts between residents	Care staff–resident
	Face masks as a physical barrier to contact with residents	Care staff–resident
	Residents prone to blame care staff for mitigation measures	Care staff–resident
	Enabling remote contact between residents and SOs	Care staff–resident–SO
	Facilitation turns from supportive to crucial	Care staff–resident–SO
	Partly taking on the role of the SO	Care staff–resident–SO
	Being the bearer of bad news towards SOs	Care staff–SO
	Facing SO’s frustration over mitigation measures	Care staff–SO
	Adhering to the mitigation measures in a strict manner	Care staff–resident–SO
The impact of COVID-19 on residents’ wellbeing	Restricted freedom for residents	
	Limited contact between residents and SOs	
	Positive effect of remote contact on residents’ wellbeing	
	Remote contact no replacement for face-to-face contact	
The impact of COVID-19 on care staff’s wellbeing	Wellbeing of care staff linked to wellbeing of residents	
	Enforcing mitigation measures leads to internal conflict	

## Data Availability

Data available on request due to privacy restrictions.

## References

[1] Dutch Central Government, Committee on Public Health, Welfare and Sports, Taskforce Infection Disease Control (2020). Report of a Technical Briefing. https://www.tweedekamer.nl/debat_en_vergadering/commissievergaderingen/details?id=2020A01496.

[2] COVID-19 Dashboard. https://coronadashboard.rijksoverheid.nl/landelijk/verpleeghuiszorg.

[3] Epidemiological Situation COVID-19 in the Netherlands, 2020. https://www.rivm.nl/sites/default/files/2020-08/COVID-19_WebSite_rapport_wekelijks_20200707_1251.pdf.

[4] Kruse F.M., Van Tol L., Vrinzen C., Van der Woerd O., Jeurissen P.P.T. The Impact of COVID-19 on Long-Term Care in the Netherlands: The Second Wave, 2020. LTCcovid, International Long-Term Care Policy Network. https://ltccovid.org/wp-content/uploads/2020/11/COVID-19-Long-Term-Care-situation-in-the-Netherlands-_-the-second-wave-25-November-2020-2.pdf.

[5] Member Poll Corona Nursing Home, 2020. https://www.venvn.nl/media/el0asgfw/01_peiling_corona_verpleeghuis.pdf.

[6] McDougall R.J., Gillam L., Ko D., Holmes I., Delany C. (2021). Balancing health worker well-being and duty to care: An ethical approach to staff safety in COVID-19 and beyond. J. Med. Ethics.

[7] McKenna H. (2020). Covid-19: Ethical issues for nurses. Int. J. Nurs. Study.

[8] Emanuel E.J., Persad G., Upshur R., Thome B., Parker M., Glickman A., Zhang C., Boyle C., Smith M., Philips J.P. (2020). Fair allocation of scarce medical resources in the time of covid-19. N. Engl. J. Med..

[9] White D.B., Lo B. (2020). A framework for rationing ventilators and critical care beds during the COVID-19 pandemic. JAMA.

[10] Nioi M., Napoli P.E., Finco G., Demontis R., Fossarello M., D’aloja E. (2021). Fear of the COVID-19 and medical liability. Insights from a series of 130 consecutives medico-legal claims evaluated in a single institution during SARS-CoV-2-related pandemic. Signa Vitae..

[11] Erdem H., Lucey D.R. (2021). Healthcare worker infections and deaths due to COVID-19: A survey from 37 nations and a call for WHO to post national data on their website. Int. J. Infect. Dis..

[12] Dutch Central Government (2021). Test Capacity Quadrupled, Corona Testing Possible for all Healthcare Personnel. https://www.rijksoverheid.nl/onderwerpen/coronavirus-tijdlijn/nieuws/2020/03/31/testcapaciteit-verviervoudigd-coronatests-mogelijk-voor-al-het-zorgpersoneel.

[13] V&VN (2020). Working without Personal Protective Equipment? Unacceptable!. https://www.venvn.nl/nieuws/werken-zonder-beschermende-middelen-onacceptabel/.

[14] V&VN (2020). V&VN poll: Masks Shortages Persist, Psychological Pressure High. https://www.venvn.nl/nieuws/peiling-v-vn-tekorten-maskers-houden-aan-psychische-druk-hoog/.

[15] Duijs S.E., Haremaker A., Bourik Z., Abma T.A., Verdonk P. (2021). Pushed to the margins and stretched to the limit: Experiences of freelance eldercare workers during the Covid-19 pandemic in the Netherlands. Fem. Econ..

[16] Vindegaard N., Benros M.E. (2020). COVID-19 pandemic and mental health consequences: Systematic review of the current evidence. Brain Behav Immun..

[17] Kruse F.M., Abma I., Jeurissen P.P.T. The Impact of COVID-19 on Long-Term Care in the Netherlands. LTCcovid, International Long-Term Care Policy Network. https://ltccovid.org/wp-content/uploads/2020/05/COVID19-Long-Term-Care-situation-in-the-Netherlands-25-May-2020-1.pdf.

[18] Litam S.D.A., Balkin R.S. (2021). Moral injury in health-care workers during COVID-19 pandemic. Traumatology.

[19] Alharbi J., Jackson D., Usher K. (2020). The potential for COVID-19 to contribute to compassion fatigue in critical care nurses. J. Clin. Nurs..

[20] Čartolovni A., Stolt M., Scott P.A., Suhonen R. (2021). Moral injury in healthcare professionals: A scoping review and discussion. Nurs. Ethics..

[21] Banks S., Cai T., de Jonge E., Shears J., Shum M., Sobočan A.M., Strom K., Truell R., Uriz M.S., Weinberg M. (2020). Practising ethically during COVID-19: Social work challenges and responses. Int. Soc..

[22] Oh N., Hong N., Ryu D.H., Bae S.G., Kam S., Kim K.Y. (2017). Exploring nursing intention, stress, and professionalism in response to infectious disease emergencies: The experience of local public hospital nurses during the 2015 MERS outbreak in South Korea. Asian Nurs Res..

[23] Van der Merwe S.E., Biggs R., Preiser R., Cunningham C., Snowden D.J., O’Brien K., Jenal M., Vosloo M., Blignaut S., Goh Z. (2019). Making Sense of Complexity: Using SenseMaker as a Research Tool. Systems.

[24] Marsick V.J., Cederholm L., Turner E., Pearson T. (1992). Action-reflection learning. Train. Dev..

[25] Braun V., Clarke V. (2006). Using thematic analysis in psychology. Qual. Res. Psychol..

[26] Glaser B., Strauss A. (1967). The Discovery of Grounded Theory: Strategies for Qualitative Research.

[27] Tronto J.C. (1993). Moral Boundaries. A Political Argument for an Ethic of Care.

[28] Tronto J.C. Protective Care or Democratic Care? Some Reflections on Terrorism and Care. Proceedings of the SIGNAL.

[29] Dichter M.N., Sander M., Seismann-Petersen S., Köpke S. (2020). COVID-19: It is time to balance infection management and person-centered care to maintain health of people living in German nursing homes. Int. Psychogeriatr..

[30] Gordon A.L., Goodman C., Achterberg W., Barker R.O., Burns E., Hanratty B., Martin F.C., Meyer L., O’Neill D., Schols J. (2020). Commentary: COVID in care homes—Challenges and dilemmas in healthcare delivery. Age Ageing.

[31] Johannessen A.K., Werner A., Steihaug S. (2014). Work in an intermediate unit: Balancing between relational, practical and moral care. J. Clin. Nurs..

[32] Sevenhuijsen S. (2000). Caring in the third way: The relation between obligation, responsibility and care in Third Way discourse. Crit. Soc. Policy.

[33] Drury J. (2018). The role of social identity processes in mass emergency behaviour: An integrative review. Eur. Rev. Soc. Psychol..

[34] Smaling H.J.A., Tilburgs B., Achterberg W.P., Visser M. (2022). The Impact of Social Distancing Due to the COVID-19 Pandemic on People with Dementia, Family Carers and Healthcare Professionals: A Qualitative Study. Int. J. Environ. Res. Public Health.

[35] Ong A.D., Bergeman C.S., Bisconti T.L., Wallace K.A. (2006). Psychological resilience, positive emotions, and successful adaptation to stress in later life. J. Pers Soc. Psychol.

[36] Huijg J.M. (2019). Personal stories improve standard care and resident wellbeing. NRC.

[37] Wanberg C.R., Csillag B., Douglass R.P., Zhou L., Pollard M.S. (2021). Socioeconomic status and well-being during COVID-19: A resource-based examination. Am. J. Appl. Psychol..

[38] Islam M.S., Baker C., Huxley P., Russell I.T., Dennis M.S. (2017). The nature, characteristics and associations of care home staff stress and wellbeing: A national survey. BMC Nurs..

[39] Coelho C.M., Suttiwan P., Arato N., Zsido A.N. (2020). On the nature of fear and anxiety triggered by COVID-19. Front. Psychol..

[40] Troisi A., Nanni R.C., Riconi A., Carola V., Di Cave D. (2021). Fear of COVID-19 among healthcare workers: The role of neuroticism and fearful attachment. J. Clin. Med..

[41] Killgore W.D.S., Taylor E.C., Cloonan S.A., Dailey N.S. (2020). Psychological resilience during the COVID-19 lockdown. Psychiatry Res..

[42] Verbeek H., Gerritsen D.L., Backhaus R., de Boer B.S., Koopmans R.T.C.M., Hamers J.P.H. (2020). Allowing visitors back in the nursing home during the COVID-19 crisis: A Dutch national study into first experiences and impact on well-being. J. Am. Med. Dir. Assoc..

[43] Verbeek-Oudijk D., Koper I. Het leven in Een Verpleeghuis: Landelijk Overzicht van de Leefsituatie, Ervaren Kwaliteit van Leven en Zorg van Oudere Verpleeghuisbewoners in 2019 [Life in a Nursing Home: National Survey of the Living Situation, Perceived Quality of Life and Care of Older Nursing Home Residents in 2019]. https://www.scp.nl/publicaties/publicaties/2021/02/19/het-leven-in-een-verpleeghuis.

[44] Dohmen M.D.W., Huijg J.H., Abma T.A., Spannring R. (2022). Democratic Care in Nursing Homes: Responsive Evaluation to Mutually Learn about Good Care. Institutions and Organizations as Learning Environments for Participation and Democracy—Opportunities, Challenges, Obstacles.

[45] CBS (2021). Mobility of Employees; Labor Market Care and Welfare (Broad), Inflow, Outflow, Balance, Region. https://azwstatline.cbs.nl/#/AZW/nl/dataset/24050NED/table?ts=1591279185724.

